# Using Electric Fields for in Situ Curing of Carbon Fiber/Phenolic Composites in Additive Manufacturing

**DOI:** 10.1021/acsaenm.5c01110

**Published:** 2026-02-24

**Authors:** Christian J. McGovern, Kyle A. Oubre, Ethan M. Harkin, Sayyam S. Deshpande, Ethan M. Walker, Carolyn T. Long, John D. Bernardin, Micah J. Green

**Affiliations:** † Artie McFerrin Department of Chemical Engineering, 14736 Texas A&M University, College Station, Texas 77843, United States; ‡ Materials Science and Technology-7, 5112 Los Alamos National Laboratory, Los Alamos, New Mexico 87545, United States; § Department of Materials Science & Engineering, Texas A&M University, College Station, Texas 77843, United States

**Keywords:** Additive Manufacturing Technologies, Electric Field Heating, In Situ Curing, Carbon Fiber Composites, Thermosets

## Abstract

In this paper, we demonstrate electrothermal heating and curing of carbon fiber (CF)/phenolic composites to enable successive deposition for additive manufacturing (aka 3D printing). Electric fields are capable of heating susceptor materials, which makes them a potential heat source for 3D printing thermoset composites, such as CF/Phenolic prepregs. We investigated the heating response of CF/phenolic prepregs when exposed to electric fields and found that our prepregs reached the target temperature of 210 °C when the electric field applicator was supplied with low power (8 W). We also show continuous heating and curing by translating prepregs through an electric field. Finally, we demonstrate additive manufacturing by manually depositing a layer or prepreg, using an electric field to perform in situ curing, and then repeating the process to create multilayer structures. This multilayer structure showed no macroscopic deformation in contrast to conventional methods and showed that additive manufacturing is possible.

## Graphical abstract

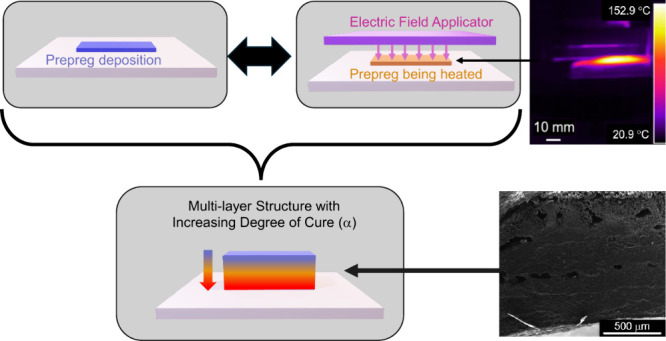

## 1 Introduction

Phenolic resins are a single-stage thermoset which are often used in conjunction with a filler (typically macroscopic carbon fiber) to form composites.^1,2^ These composites are known for their mechanical strength, chemical resistance, and high degradation temperature.^2–8^ As a result, phenolic/CF composites are often used in aerospace, automotives, and construction.^2,9–12^ In many of these applications, phenolic resin is pyrolyzed to form carbon–carbon composites.^11,13–15^

Void formation is a common problem that occurs when curing phenolic resin.^16^ Unlike many other common thermoset curing processes, the phenolic curing reaction produces large quantities of byproducts that are emitted as vapor.^16^ These vapors often form closed gas pockets, referred to as voids, because the vapors become trapped during curing and gelation.^16–22^ Such voids are naturally associated with decreased mechanical strength and thermal conductivity as well as a source for crack initiation.

The most common method for processing phenolic composites is to compress mold prepregs (partially cured composites) into desired shapes. The first step of this process is infiltrating a filler preform with phenolic resin. The composite then undergoes B-staging (partial curing) to form a prepreg. B-staging aids in the removal of byproducts which reduces the degree of void formation and provides a malleable starting material.^23–26^ The prepregs are then cut to size and layered upon one another in a mold. The layered prepregs are then compression molded at pressures reaching up to 20,000 psi and heat bonded together (typically 130 – 200 °C) to form a cured composite part.^16,27^ The combination of B-staging and high pressures eliminate macroscopic voids; however, it is still possible for trapped vapors to form micro voids.^22,27,28^ A primary problem with compression molding is the significant capital required due to machinery, mold, and operational costs.^28,29^ Compression molding has limits on the complexity of the geometrical structures that can be produced. Because additive manufacturing builds structures layer by layer, it is far less limited in the ability to make complex three-dimensional structures.

We have previously studied the entrapment of vapors in phenolic resins during curing; we found that if a phenolic composite is sufficiently thin, the byproducts of the curing reaction can diffuse and escape.^29^ We also found that high cure speeds (associated with high cure temperatures) cause the entrapment of byproducts which form voids in bulk samples.^29^ These findings indicate that thick phenolic samples possess voids when cured quickly; however, a thin sample avoids these issues.^29^ This work also indicates that multilayer structures can be built by performing in situ curing of thin samples, which would alleviate void formation.

With this in mind, we propose the simultaneous additive deposition of thin CF/phenolic prepregs and in situ curing to create fully cured composites, eliminate void formation, reduce capital costs, and enable rapid prototyping. Capital costs associated with equipment and molds are greatly diminished with additive deposition. Additionally, additive deposition allows for the creation of multifunctional structures in complex geometries. The simplest method for additive deposition is to deposit successive layers of prepregs onto a substrate followed by a post cure; however, this method is not feasible since curing thick phenolic samples results in voids as discussed above.^29^ We hypothesize that by using single-layer deposition and in situ curing, each layer of the manufactured structure will be thin, which reduces the distance that volatiles must travel to be released. By allowing the volatiles to escape each layer, we can hypothetically perform in situ curing at high-temperatures without macroscopic deformation. We chose to use prepregs as our starting material because prepregs have already evolved some of the reaction byproducts during B-staging.

To incorporate thermoset composites as feedstock materials for additive manufacturing, the capability to build self-supporting structures is required. To create self-supporting structures, a method of providing in situ curing is needed. Prior methods of in situ curing have primarily focused on frontal polymerization.^30–32^ Few papers have explored the usage of external heating sources to continuously cure thermoset composites during additive deposition.^33,34^

Electric fields provide a promising avenue for in situ heating, which eliminates the need for an oven or autoclave and further reduces capital costs. Radio frequency (RF) fields and dielectric barrier discharge (DBD) are two types of electric fields which have been used for additive manufacturing by our group.^35,36^ These electric fields have been shown to be useful for additive manufacturing and produce significant heating responses in susceptor materials such as CF and carbon nanotubes.^35,36^

Here, we demonstrate the use of electric fields to cure in situ carbon fiber/phenolic composites during successive deposition. We develop a cure schedule to produce suitable prepregs and analyze the byproducts removed during B-staging. We use electric fields to heat prepregs to a target temperature. We demonstrate that electric field heating enables the continuous in situ curing of CF/Phenolic prepregs. Lastly, we perform additive deposition by creating multilayer structures using electric fields and in situ curing.

## 2 Materials and Methods

### 2.1 Materials and Prepreg Production

Plenco 14670, a resole phenolic resin, was donated by Plenco and was used in the production of all samples. The T700S carbon fiber spool used to manufacture our samples was purchased from Toray. Unidirectional prepregs were produced by infiltrating T700s carbon fiber tows with Plenco 14670, doctor blading the samples, and then performing B-staging in an oven for 10 min at 110 °C. These prepregs are approximately 7 mm wide and 0.15 mm thick, and they are approximately 52% carbon fiber by weight.

### 2.2 Malleability Testing of Prepregs

Prepregs with varying degrees of cure were produced by varying the cure time during B-staging. An oven was set to a temperature of 110 °C. Once the temperature reached 110 °C, carbon fiber tows infiltrated with uncured phenolic were heated for various times (10, 15, 20, and 25 min) to form prepregs. The prepregs were manually deformed at a 90° angle and then released to observe malleability and shape retention.

### 2.3 TGA Analysis

Thermogravimetric analysis (TGA) tests were performed in air with a temperature ramp of 20 °C per minute. One test proceeded to 350 °C at which time no further mass loss occurred due to curing. The second test ramped the temperature to 110 °C and then held the system isothermally for 10 min to recreate prepreg production.

### 2.4 RF Apparatus and Heating

RF signals of a user-specified frequency were generated using a RIGOL DSG15 model signal generator. The RF signal then passed through a PRANA GN 500 power amplifier to amplify the signal to a target power.^35^ Lastly, the RF signals were delivered to an RF applicator, which projects an RF field between two copper traces (Figure S1). The amplifier and RF applicator were placed inside of a handmade faraday cage consisting of brass mesh, and the connections between these pieces of equipment consisted of 50 Ω coaxial cables to ensure safe operation. The heating response for the RF experiments were recorded using a FLIR A600 model infrared camera and the accompanying FLIR ResearchIR software.

A Vector Network Analyzer (VNA) was utilized to measure the S11 parameter of the RF applicator in the range (100–200 MHz). The VNA was connected to the y-applicator, and the S11 parameter was measured without a load. A prepreg was then placed between the copper traces of the applicator, and the S11 parameter was measured again over the same frequency range.

RF heating of carbon fiber/phenolic preparations was investigated. Prepregs were placed between the two copper traces of the RF applicator. A frequency sweep test was conducted where the sample was heated at a given frequency and 1 W for 1 s followed by 13 s of cooling.^37^ After cooling, the frequency would increase by 1 MHz, and the sample would again be heated for 1 s before cooling. This test procedure was repeated until all frequencies in the range of 1 to 200 MHz were tested. The frequency was then set to 120 MHz and the power was modulated to maintain a temperature of 210 °C for 2 min to fully cure the composites.

### 2.5 DBD Apparatus

The Dielectric Barrier Discharge (DBD) mobile electric field applicator consists of two electrodes arranged vertically and separated by a dielectric barrier and an air gap.^38^ The dielectric barrier in this apparatus is a ceramic disk that is approximately 70 mm in diameter and 3 mm thick, while the air gap is adjustable. To generate the electric field, the DBD receives an input of 24 V DC which is then pulse width modulated to create a square wave and a variable duty cycle. This is then combined with a 37.7 kHz carrier signal to create high frequency pulses which manifest as plasma filaments.^39^ The power of the electric field is controlled by modulating the duty cycle, which determines how often the plasma is allowed to form. When the duty cycle is increased (0–100%), the time between pulses is shortened; thus, plasma is formed more frequently. When a conductive sample is placed between the two electrodes, the plasma discharges through the sample, which causes heating.

### 2.6 Continuous Curing of Prepregs

Long prepregs were prepared by infiltrating 30 in.-long carbon tows with phenolic before wrapping the composites around a glass stirring rod. The rod and composites were then placed in an oven at 110 °C for 10 min to form prepregs. The prepregs were then wrapped around a spool with wax paper placed between the wound layers to prevent sticking. The wound prepregs were then pulled through an electric field by a syringe pump at a rate of 0.225 mm per second.

### 2.7 Producing Multilayer Structures

Prepregs (60 mm long) were manually deposited on an aluminum build plate and partially cured by an electric field (150 °C for 150 s). Note, the power is varied to maintain the desired temperature, and the build plate was not temperature controlled. Then, prepregs (30 mm in length) were manually deposited upon the first layer and partially cured (150 °C for 150 s). This process was repeated to create a ten-layer structure. Separate multilayer structures were created by stacking ten prepregs onto one another and curing in an oven.

### 2.8 Scanning Electron Microscopy

The ten-layered structures created in Section 2.7 were submerged in liquid nitrogen. The samples were then cryofractured to reveal the cross sections, and the cross sections were polished. Using Quanta SEM, cross section images were taken for the in situ-cured and oven cured structures.

### 2.9 Lap Shear Testing

Prepregs (made at 110 °C for 600 s) were heated at 120 °C for 0, 225, 425, 650, and 900 s in an oven to create composites with varying degrees of cure (34, 50, 60, 70, and 80%). A second set of prepregs were placed on the first with an overlapping area of 0.25 cm^2^. The structures were then fully cured in an oven at 120 °C and subjected to lap shear testing using an MTS Insight tensile and compression testing machine.

## 3 Results and Discussion

### 3.1 Key Concept

This work demonstrates the use of electric fields for continuous, in situ heating of phenolic composites for additive manufacturing. Figure 1 depicts the in situ curing process for multilayer structures. The process involves depositing prepregs, while a mobile electric field applicator heats the material, increasing its degree of conversion (α). The top layer undergoes the highest heating rates when placed in the electric field; however, the bottom layers will also continue to heat due to conductive heat transfer.^35^ We hypothesize that additively depositing a structure using prepregs and in situ curing reduces void formation and makes structures without deformation. To evaluate this hypothesis, we first investigate electric field heating of phenolic prepregs followed by the use of electric fields during additive deposition.

**Figure 1:**
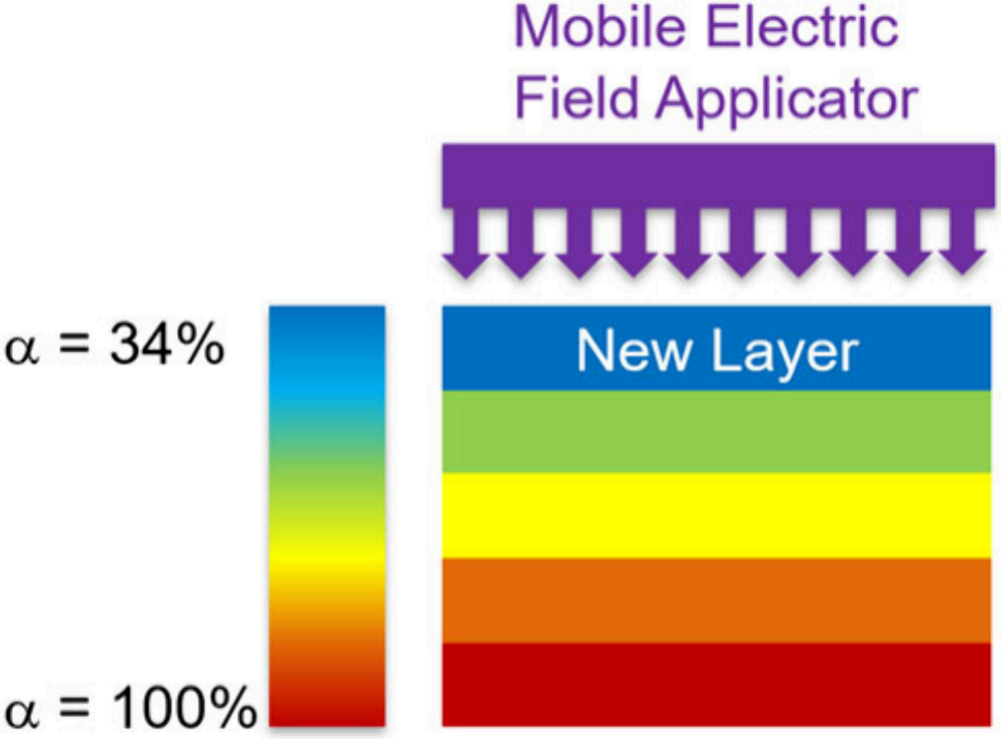
Schematic showing the in situ curing process of carbon fiber/phenolic prepregs. A new layer of prepreg (α = 34%) is deposited before an electric field applicator is positioned over the structure. The electric field heats the freshly deposited layer as well as several underlying layers, causing the degree of conversion (α) to increase with every pass of the applicator.

### 3.2 Prepreg Preparation

First, a suitable cure schedule is needed to produce prepregs for additive deposition. The ideal prepreg needs to be tacky enough to adhere to a substate, while malleable enough to deform at a 90° angle while being extruded from a printer. A low degree of conversion (alpha ∼ 30%) is associated with malleability and tack, which are necessary for deposition, but a high degree of conversion (alpha ∼ 90%) preremoves volatiles from the system. Thus, the highest alpha that maintains malleability is ideal. Note that we can relate a cure schedule to a degree of cure by using the kinetic model developed by Liang et al. (Table S1).^40^ Liang et al. developed this model by performing DSC on a resole phenolic resin. Figure S2 shows the model prediction for cure kinetics at varying temperatures. A temperature of 110 °C was chosen to vaporize water (a primary component of the evolved byproducts), while also allowing for slow curing. Slow curing enables very precise control over the degree of conversion of the prepregs. Having chosen a cure temperature, we still needed a cure time to create our cure schedule. We produce various prepregs in the laboratory by varying the cure time at the chosen temperature of 110 °C. After curing, the prepregs are then manually deformed at a 90° angle and released to investigate how an increasing degree of conversion diminishes malleability (Figure S3). This malleability test showed that cure times of 10 and 15 min produced prepregs suitable for additive manufacturing; we selected 10 min as the cure time.

We used TGA analysis to quantify the release of byproducts during prepreg formation and the total mass loss of the curing reaction. We conducted two TGA experiments on neat phenolic in air with a ramp rate of 20 °C/min (Figure 2). In Figure 2A, the temperature is ramped to 350 °C and fully cures the phenolic. In Figure 2B, the temperature is ramped to 110 °C and then undergoes an isothermal hold for 25 min to recreate candidate cure schedules for prepreg production. The degrees of conversion of the samples were calculated using the kinetic model developed by Liang et al. for phenolic curing.^40^ Note that the curing kinetics cannot be measured by DSC because of the vapors produced during the reaction. However, Figure 2A shows that the mass loss ceases at nearly the same time that the model predicts complete curing. This indicates that the model closely approximates the degree of conversion. Table 1 provides a comparison of the residual masses and degrees of conversion for the cure schedules. Table 1 also includes the percent reduction of byproducts during prepreg formation. One can observe from the TGA data that most of the byproducts are released during the early stages of the curing reaction. Our chosen cure schedule of 110 °C for 10 min evolved a large fraction (73.0%) of the total byproducts generated by the curing reaction.

**Figure 2:**
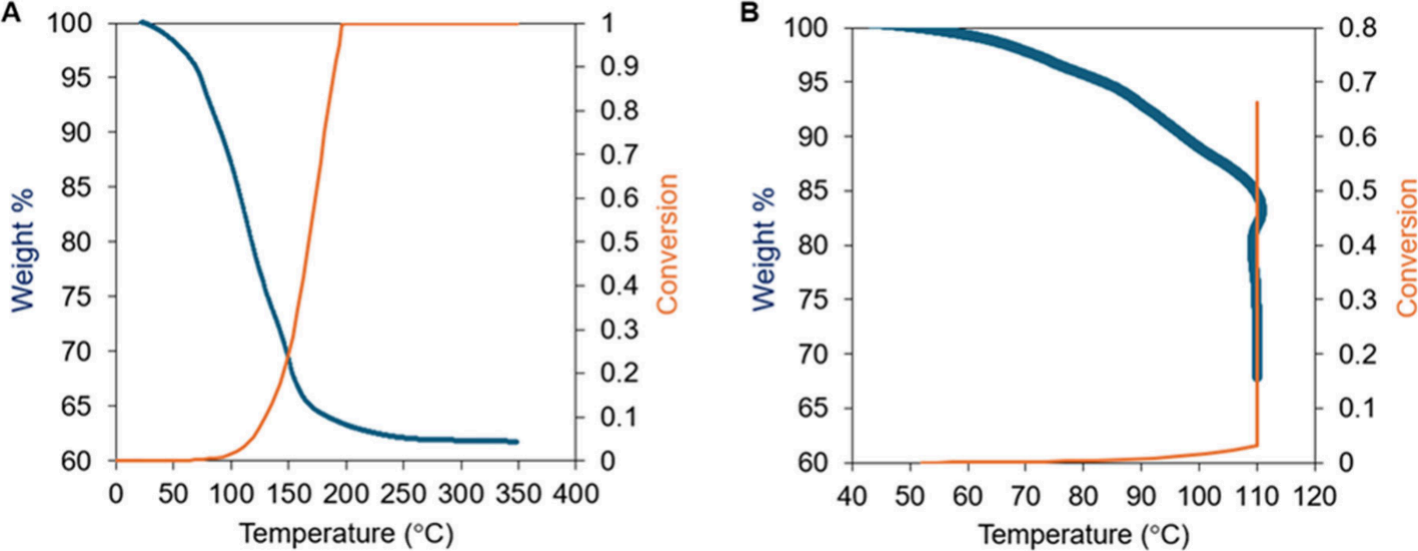
TGA of neat phenolics in air with a temperature ramp of 20 °C/min. The degree of conversion of the phenolic is calculated using the kinetic model developed by Liang et al.^40^ (A) In this case, the temperature ramp proceeds until 350 °C and shows the total mass lost during curing. (B) In this case, the temperature ramp proceeds until 110 °C, where it is then held isothermally for 10 min.

**Table 1:** Analysis of TGA Data (Figure 2B) to Aid in the Selection of Prepreg Cure Schedule (20 °C/min Ramp to 110 °C then Hold Time)^a^

Cure Time at 110 °C	Weight %	% Byproducts Evolved	Degree of Conversion
10 min	72.08	73.04	0.3365
15 min	70.04	78.37	0.4630
20 min	68.74	81.78	0.5702
25 min	67.88	84.03	0.6626

a Cure schedules are recorded with their corresponding sample weight, percent reduction of byproducts, and the degree of conversion, which is calculated from the model.

### 3.3 RF Response of Prepregs

We next examine the heating of carbon fiber/phenolic prepregs in response to RF fields. In prior work from our group, RF fields have been used to heat prepregs and bond composites and repair damaged zones on composites. In addition to showing that susceptors heat in response to RF fields, these works also show that susceptors heat most efficiently at certain frequencies.^37,41,42^ We used a VNA (Visual Network Analyzer) to analyze the S11 parameter (reflection coefficient) of the RF applicator over a range of frequencies (100–200 MHz). Figure 3A shows that S_11_ is minimized at a frequency of 125 MHz. Additionally, we can use the S_11_ value at this frequency to calculate the percentage of power reflected (11.5%) and the percentage of power being coupled to the sample (88.5%). We next determine the frequency at which maximum heating occurs by exposing the prepreg to electric fields of varying frequencies. Figure 3A also shows that carbon fiber/phenolic composites heat in the presence of RF fields, and it provides heating rates for frequencies in the range 100–200 MHz, with maximum heating occurring at 120 MHz. Note that the VNA analysis and the heating analysis yield similar values for the desired frequency, as expected.

**Figure 3:**
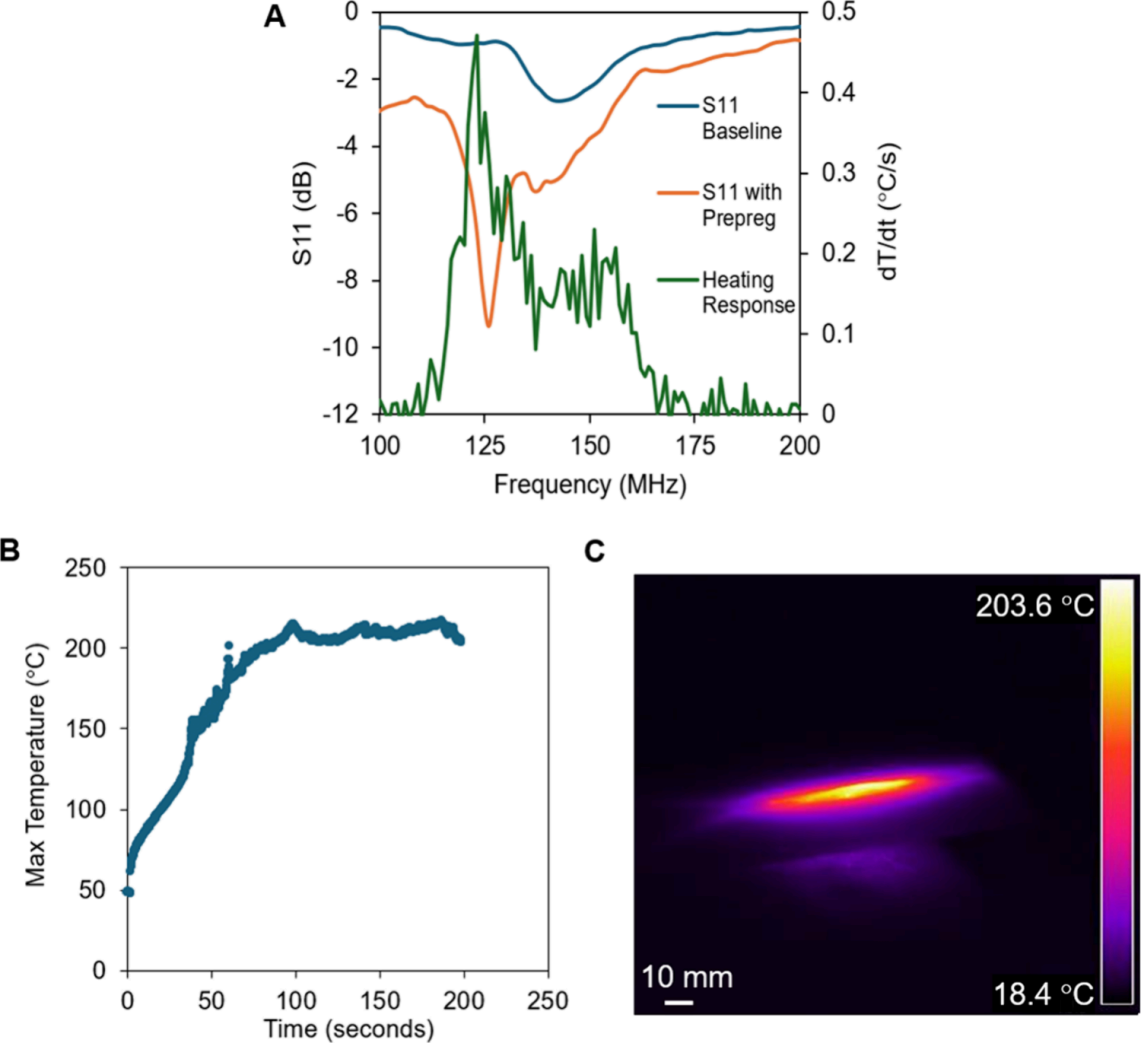
(A) S_11_ versus frequency for the electric field applicator measured with a VNA (with and without a prepreg) and the observed experimental heating rate (based on change in maximum temperature) as a function of frequency for a carbon fiber/phenolic prepreg (applied RF power = 1 W). (B) Maximum temperature versus time data for RF curing of the prepreg (frequency = 120 MHz, RF power was modulated to achieve target T of 200 °C). (C) A thermal image of the carbon fiber/phenolic prepreg was taken while the sample heated in response to an RF field (same as in B).

### 3.4 Curing Prepregs with RF Fields

We next aim to heat and cure prepregs at a target cure temperature using electric fields. To cure stationary prepregs, the frequency of the electric field was set to 120 MHz and the power was modulated to maintain a sample temperature of 210 ± 5 °C for 2 min. Figure 3C shows the maximum temperature versus the time during curing. Figure 3D shows a thermal image of the prepreg during curing and demonstrates the heating provided by the electric field. Figure S4 displays the resulting cured composite with no observable deformation. The data suggest that single-layered samples can indeed be cured without deformation.^40^

### 3.5 Continuous Curing Using DBD

To demonstrate the ability to continuously cure prepregs in an additive manufacturing context, we carried out the following study: we used a Dielectric Barrier Discharge (DBD) applicator (70 mm in length) to cure moving spools of prepreg (0.225 mm/second) to obtain a residence time of 311 s. The kinetic model indicates full cure for this thermal schedule (150–200 °C).^40^ The DBD applicator is utilized for this experiment because it can be integrated into 3D printers or fiber deposition equipment, as shown in our prior work, without any disruption of the printer electronics.^36,39,43^ In this prior work, we demonstrated that DBD plasma heating has a linear relationship with duty cycle (power) as expected and a nonmonotonic relationship with filler loading, with a particular loading (and conductivity) that results in maximum heating.^39^

Here we demonstrate that DBD heating enables the rapid, continuous curing of phenolic prepregs. Figures 4A and 4B show images of the experimental apparatus. Figure 4C shows the maximum temperature versus the time during heating. We observe a fast initial heating rate (14 °C/s) to 150 °C by applying a duty cycle of 80%. This fast initial heating rate is observed because power is delivered directly to the sample via a high-voltage plasma.^43^ The DBD can produce a heating response without frequency-dependent impedance matching. As the sample passes through the DBD, a cured composite (90 mm in length) is produced (Figure 4D). This finding confirms that in situ curing can be conducted in a continuous, roll-to-roll manner.

**Figure 4:**
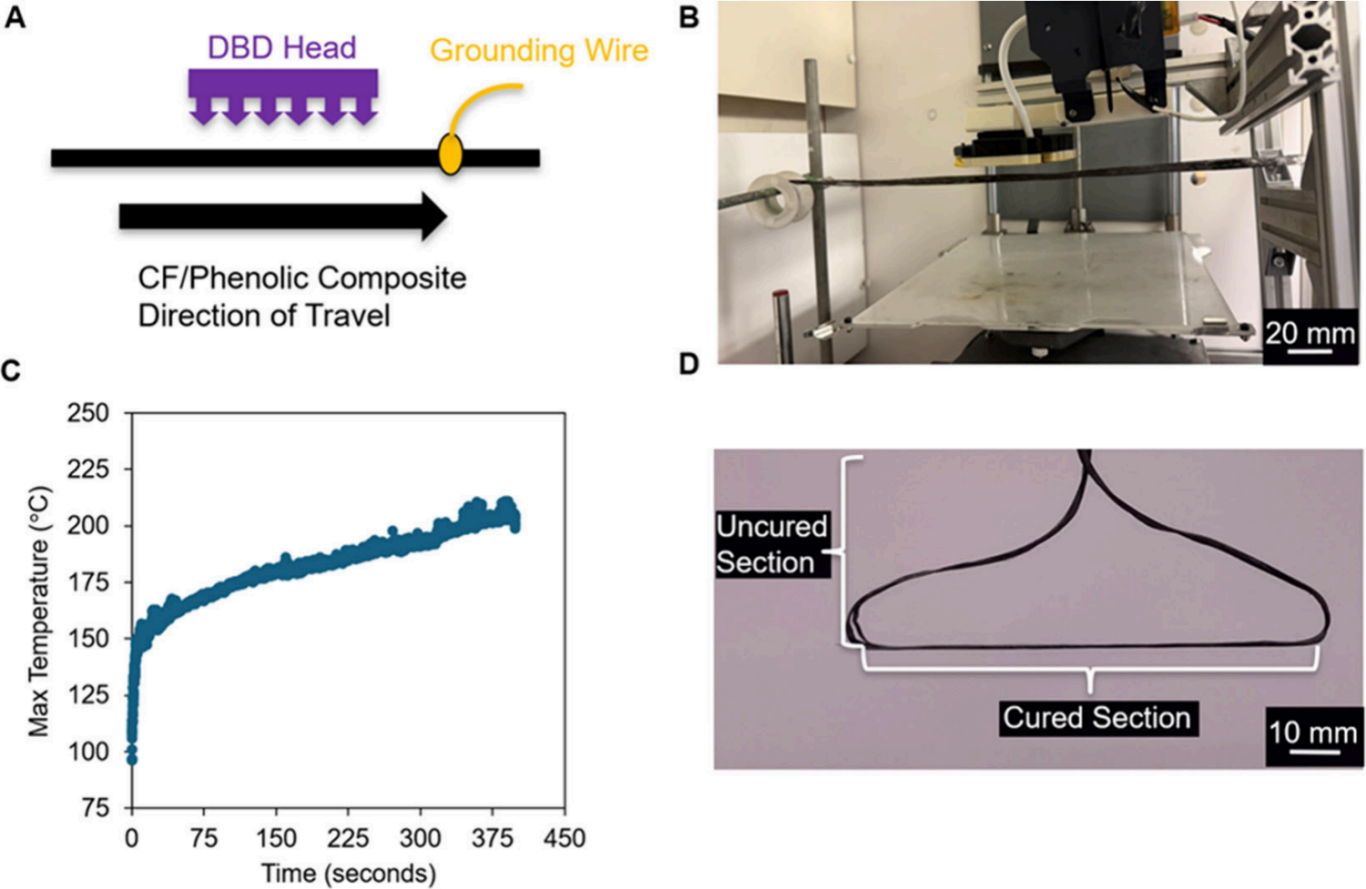
(A) Diagram of the in situ curing experimental set up. (B) Image of the experimental apparatus. (C) Experimental maximum temperature versus time data (duty cycle = 80%, potentiometer = 80). (D) A fully cured section of composite being suspended by the prepreg section.

### 3.6 Creating Multilayered Structures

We next used thin prepregs and in situ curing to perform additive deposition and create multilayer structures. We create multilayer structures by repeatedly depositing prepregs onto a build plate and performing partial, in situ curing using an electric field produced by the DBD applicator. The cycle of deposition and in situ curing is repeated for 10 layers. Note that the first layer is longer than the following layers to provide a surface for electrical grounding. Figure 5A illustrates the experimental setup, and Figure 5B shows the predicted degree of cure after being exposed to the electric field, assuming a uniform temperature. We treat the sample as thermally uniform because it is sufficiently thin in the *z*-direction. Figure 5C shows an image of the first layer (approximately 0.1 mm thick) being heated. Note that the visible “hot zone” appears larger than the sample because the sample is between two reflective surfaces (the aluminum substrate and the Kapton tape on the DBD head). Power was controlled in this experiment to maintain a temperature of 150 °C for 150 s for all layers to provide a 70% degree of cure (Figure 5D). A degree of cure of 70% was chosen because lap shear data show that this degree of conversion provides good interlayer adhesion (Figure S5). By obtaining this degree of cure, we remove most volatiles from the material which minimizes the potential for void formation at the interlayer interface. This is done while still allowing for cross-linking with the succeeding layer. Note that voids at the interlayer interface are problematic because they reduce the bonded area and serve as stress concentrators.

**Figure 5:**
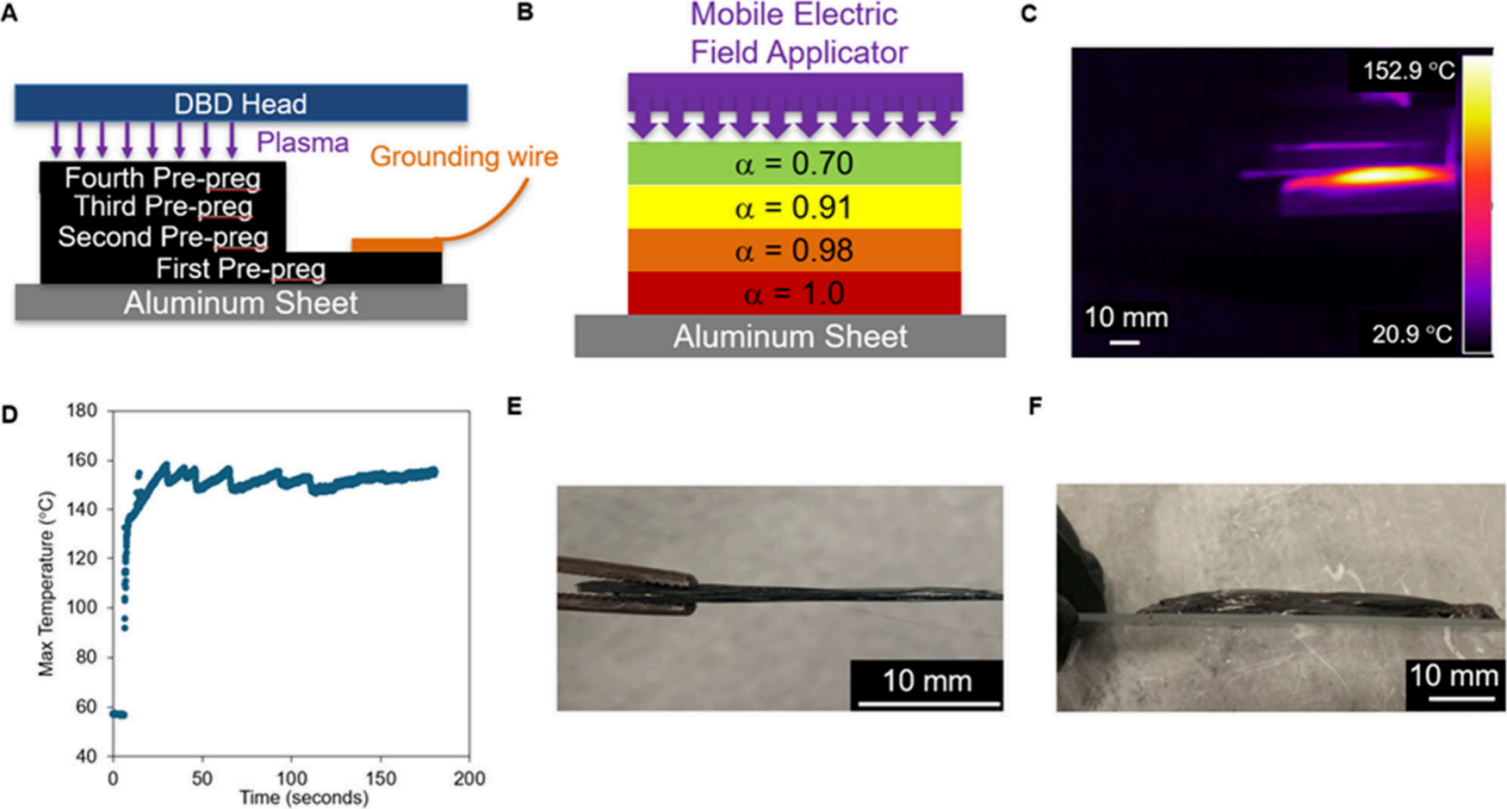
(A) Diagram showing the in situ curing used in the creation of the DBD cured, multilayered structure. (B) Depiction of the multilayer structure showing the degree of cure of the first four layers. The degree of cure is calculated using the chosen cure schedule (150 °C for 150 s for each layer) and the kinetic model.^40^ (C) Thermal image of a single layer (approximately 0.1 mm thick) heating (duty cycle modulated to maintain target temperature of 150 °C). (D) Experimental maximum temperature versus time data for a single layer. (E) Side view image of the 10-layered structure (approximately 1 mm thick) cured in situ using DBD which shows no macroscopic deformation. (F) Side view image of a multilayered structure postcured in an oven which shows significant macroscopic deformation.

We visually analyze the multilayer structures to determine whether in situ curing eliminates macroscopic deformation and compare them to structures made without in situ curing. Figure 5E is a picture of the resulting multilayer structure which does not possess noticeable deformations. This confirms that using thin prepregs in combination with in situ curing can prevent macroscopic deformation. We then create a separate multilayer structure to serve as a comparison. This structure was produced by stacking 10 prepregs onto one another and curing them in an oven without in situ curing. Figure 5F presents an image of the oven-cured structure, which showed significant macroscopic deformation, evident by the swelling of the sample. The comparison of Figures 5E and 5F confirms that in situ curing is necessary for the additive manufacturing of phenolic composites to create multilayer structures.

### 3.7 SEM of Cross Sections

To show that in situ curing produces phenolic composites without deformation, the structures shown in Figures 5E and 5F were imaged using SEM Figure 6A shows the cross section of the structure cured in situ using DBD. The structure appears to have a predominant phenolic matrix, with some voids occurring. In Figure 6B, we see clearly that the top of the sample is more porous than the bottom of the sample. It is incredibly likely that the successive addition of layers causes resin to fill the pores of the underlying layers. Thus, the bottom layers have been densified by additive deposition, while the top layers have not been infilled. Figure 6C shows a void approximately 80 μm wide. Although some voids are present, such as the one shown in Figure 6C, the surrounding area is dense. Figure 6D is the cross section of the oven cured structure, which shows large voids on the scale of millimeters instead of microns. Figures 6E and 6F show one of the relatively dense areas of the oven cured structure. Overall, by examining Figures 6A–F, we see that in situ curing drastically reduces the deformation and void formation. These SEM images show a significant contrast between the samples in terms of void formation; additional quantitative void analysis was not carried out.

**Figure 6:**
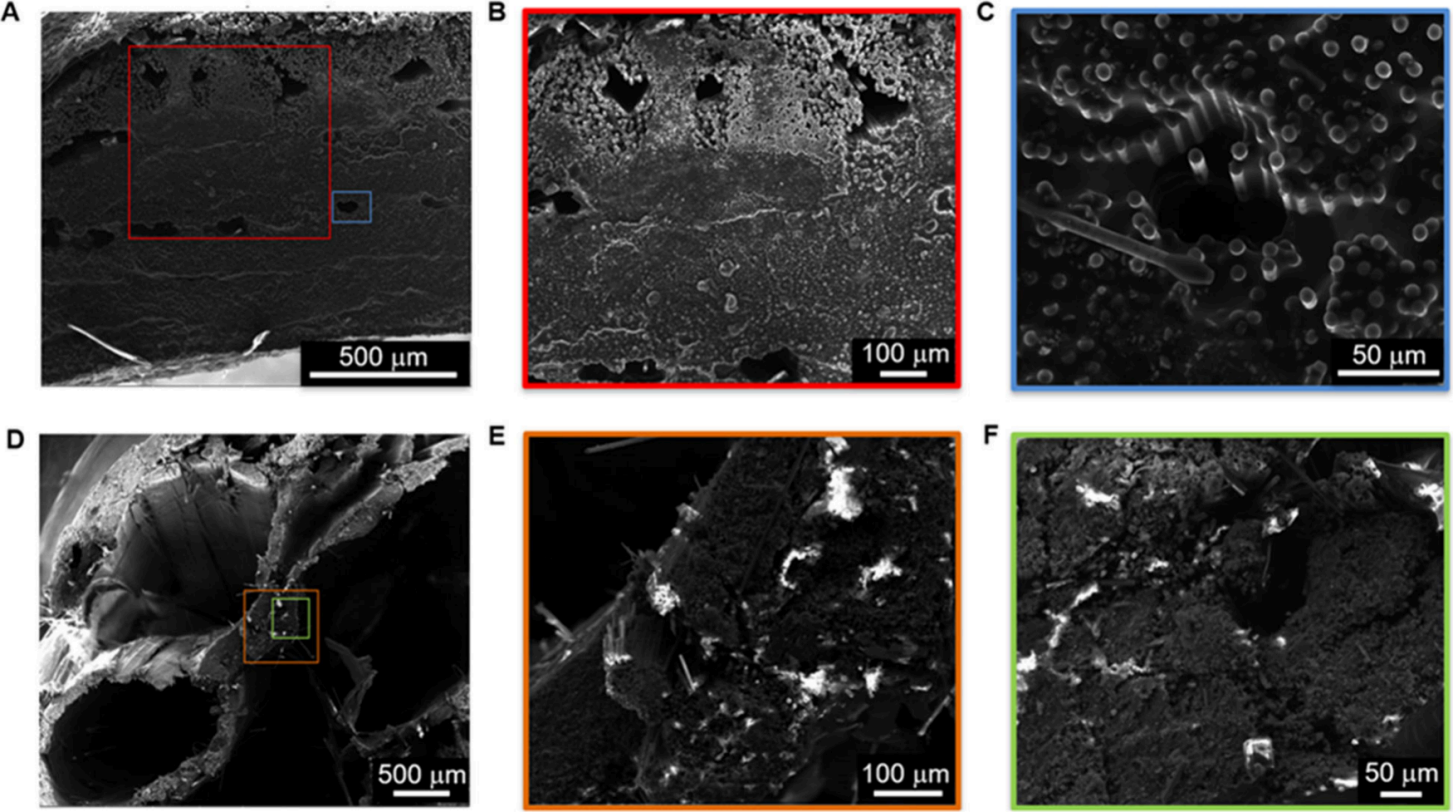
SEM Images with color matching boxes. (A–C) Cross section of structure cured in situ with DBD. (D–F) Cross section of structure cured in oven.

## 4 Conclusions

This work demonstrates the electric field heating response and in situ curing of CF/phenolic composites for their potential use in additive manufacturing. RF fields were found to be capable of quickly heating and curing CF/phenolic composites at elevated temperatures. Additionally, the continuous curing and multilayer experiments show the feasibility of electric field heating for enabling the use of thermoset composites in an additive manufacturing environment. The continuous curing experiments show that the extruded material can be cured in a continuous roll-to-roll manner. The comparison between Figures 5E and 5F shows that in situ curing is not only feasible but necessary for preventing voids since structures created without in situ curing (Figure 5F) show significant deformation. This claim is further validated by comparing Figures 6A and 6D. Deployment in a full scale 3D printer would require additional engineering and automation for depositing prepregs and modulating the electric field to obtain target temperatures.

## Supplementary Material



## Data Availability

Data will be made available on request.

## Notes

Schematic of RF apparatus, kinetic model predictions for various cure times and temperatures, table listing kinetic model parameters Liang et al.,^40^ malleability tests on prepregs, image of CF/phenolic composite cured with RF, and lap shear strength of composites formed from various cure schedules (PDF)
