# Supplementary material for: Using Electric Fields for in Situ Curing of Carbon Fiber/Phenolic Composites in Additive Manufacturing
Source: ACS Appl Eng Mater. 2026 Feb 24;4(3):1389–97. doi: 10.1021/acsaenm.5c01110 (PMC13036710; doi:10.1021/acsaenm.5c01110)
Supplement: Supplementary file 1 [file em5c01110_si_001.pdf]

Supporting Information

Using Electric Fields for In-Situ Curing of Carbon Fiber/Phenolic Composites in Additive Manufacturing

Christian J. McGovern[a], Kyle A. Oubre[a], Ethan M. Harkin[a], Sayyam S. Deshpande[a], Ethan M. Walker[b], Carolyn T. Long[b], John D. Bernardin[b], Micah J. Green[a,c*]

[a]Artie McFerrin Department of Chemical Engineering, Texas A&M University, College Station, TX, 77843, USA

[b]Materials Science and Technology-7, Los Alamos National Laboratory, Los Alamos, NM, 87545, USA

[c]Department of Materials Science & Engineering, Texas A&M University, College Station, TX, 77843, USA

* corresponding author: micah.green@tamu.edu

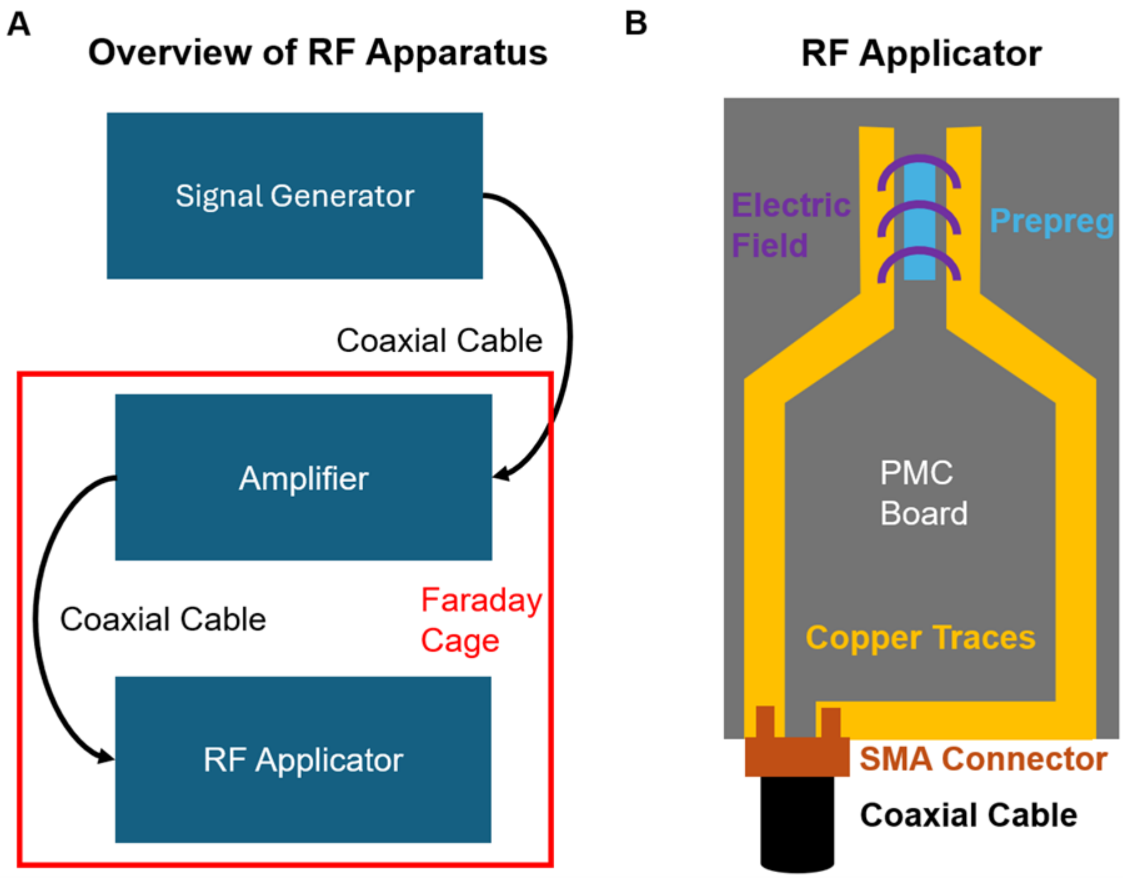

Figure S1: (A) Diagram depicting the setup of the RF apparatus, (B) Diagram showing the RF applicator and its various components

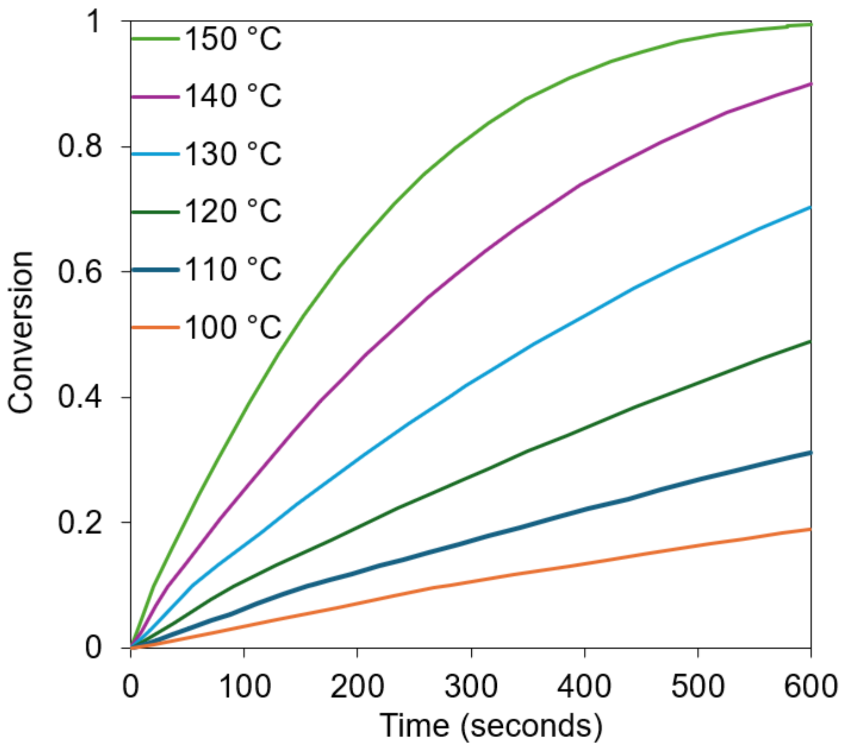

Figure S2: Kinetic model predictions were made for the degree of cure vs. time for phenolic resin using the model obtained from Liang *et al.*[1]

Autocatalytic Model: $d\alpha/dt = k(\alpha^m)(1-\alpha)^n$			Arrhenius Equation: $k = Aexp(-Ea/RT)$	
Degree of Cure (α)	m	n	Ea (kJ/mol)	A (sec^{-1})
$\alpha < 0.1$	0.111	1.7763	67.73	1.7468×10^6
$\alpha < 0.4$	0.111	0.8175	67.73	1.2031×10^6
$\alpha < 1$	0.111	0.7575	67.73	1.1448×10^6

Table S1: The kinetic model for calculating degree of cure (α) of phenolic resin with respect to time (t) from Liang *et al.*[1] The table contains the kinetic parameters (m and n), the activation energy (E_a), and Arrhenius factor (A) for each segment of the curing reaction.

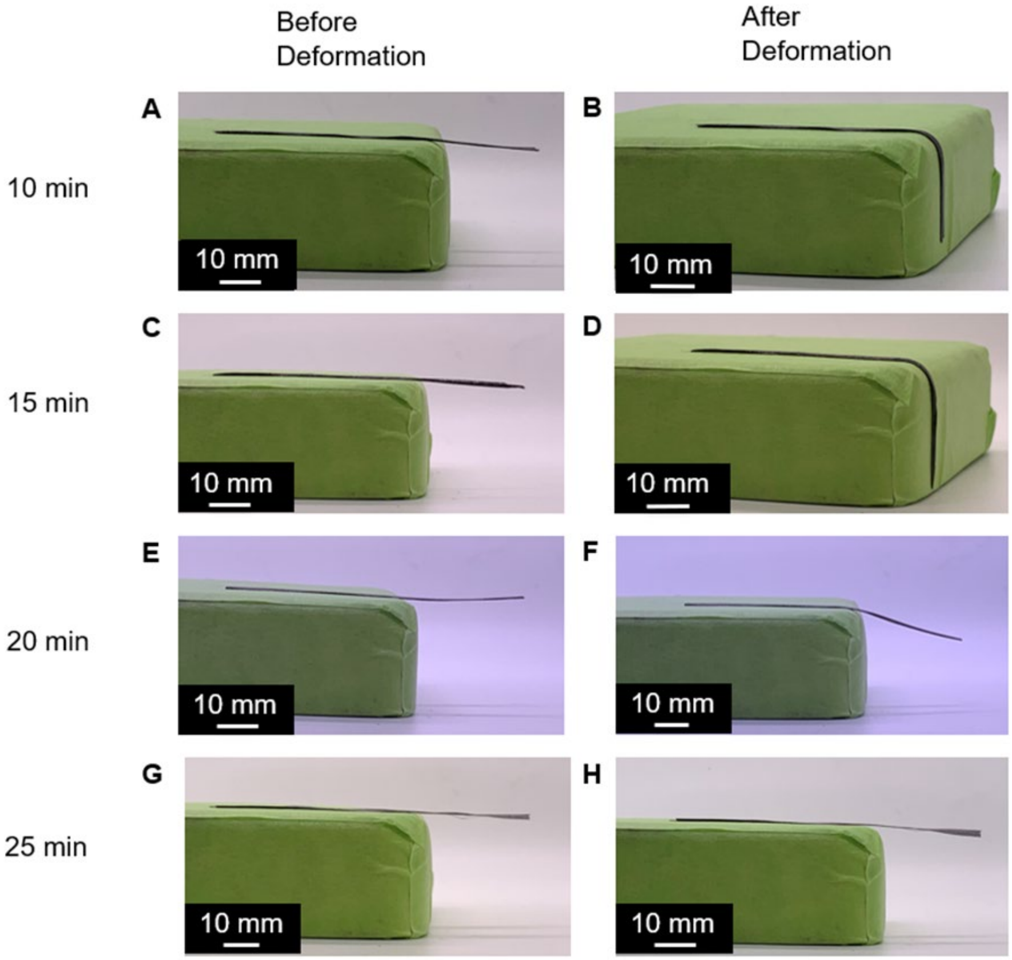

Figure S3: Sample cured at 110 °C for 10 minutes, before (A) and after (B) deformation; Sample cured at 110 °C for 15 minutes, before (C) and after (D) deformation; Sample cured at 110 °C for 20 minutes, before (E) and after (F) deformation; Sample cured at 110 °C for 25 minutes, before (G) and after (H) deformation. While both 10 and 15 minutes were observed to be acceptable cure times, a time of 10 minutes was chosen.

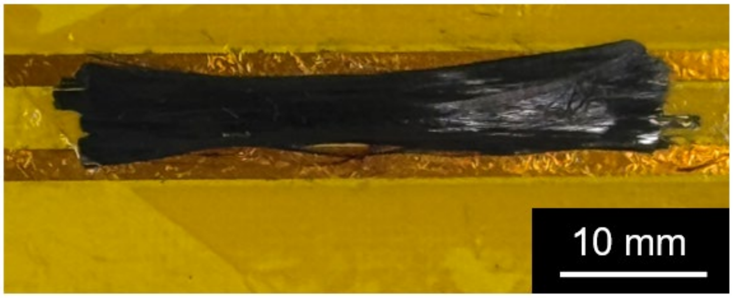

Figure S4: Digital Image of the fully cured carbon fiber/phenolic composite which resulted from the heating shown in **Figure 3**.

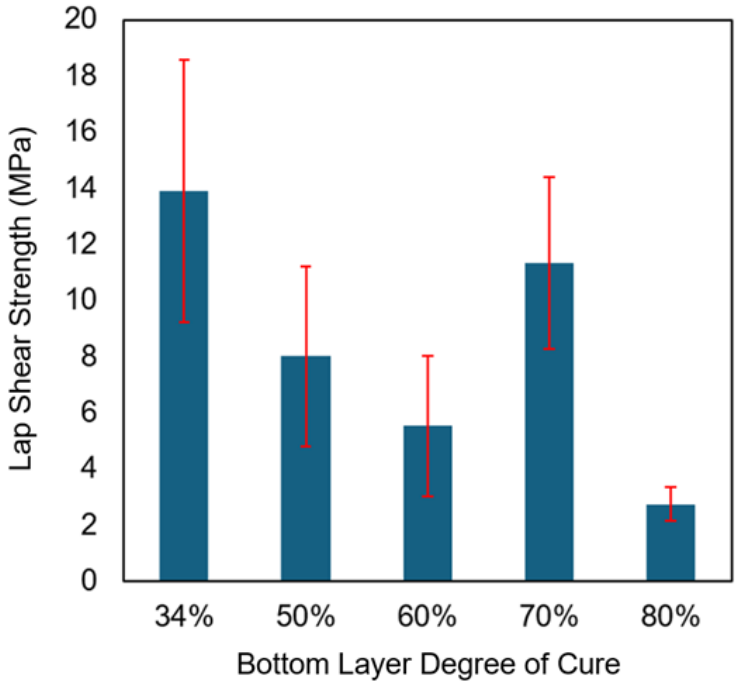

Figure S5: Lap shear data of cured composite structures. Prepregs with various degrees of cure were made by controlling cure time. These prepregs formed the first layer (bottom layer). A second set of prepregs (34% degree of cure) were laid upon the first set (overlapping area was 0.25 cm^2). The structures were then fully cured and lap shear testing was performed.

REFERENCES

1. Liang, Q., Hou, X., Feng, X., Zhang, K. & Li, J. Reaction model and cure kinetics of fiber-reinforced phenolic system. *Acta Mechanica Sinica/Lixue Xuebao* **38**, (2022).
